# Have we been qualifying measurable residual disease correctly?

**DOI:** 10.1038/s41375-023-02026-4

**Published:** 2023-09-13

**Authors:** Yahui Feng, Saibing Qi, Xueou Liu, Li Zhang, Yu Hu, Qiujin Shen, Xiaowen Gong, Wei Zhang, Junxia Wang, Wen Yan, Tiantian Wang, Huijun Wang, Zhen Song, Xiaofan Zhu, Robert Peter Gale, Junren Chen

**Affiliations:** 1 State Key Laboratory of Experimental Hematology, National Clinical Research Center for Blood Diseases, Haihe Laboratory of Cell Ecosystem, Institute of Hematology & Blood Diseases Hospital, Chinese Academy of Medical Sciences & Peking Union Medical College, Tianjin, China; 2 Tianjin Institutes of Health Science, Tianjin, China; 3 Centre for Haematology, Department of Immunology and Inflammation, Imperial College of Science, Technology and Medicine, London, UK

**Keywords:** Acute lymphocytic leukaemia, Paediatrics

*Someone told me that each equation I included in the book would halve the sales. I therefore resolved not to have any equations at all. In the end, however, I did put in one equation, Einstein’s famous equation, E = m c squared. I hope that this will not scare off half of my potential readers*.

Stephen Hawking

## Introduction

There is considerable interest in tests quantifying remaining leukaemia cells after therapy, termed measurable residual disease (MRD)-tests, to predict therapy outcomes, leukaemia recurrence and consider potential subsequent interventions [1–10]. Many studies reported a negative MRD-test during or after completing anti-leukaemia therapy independently identifies persons with a low risk of leukaemia relapse compared with those with a positive MRD-test after adjusting for other predictive and prognostic co-variates [5, 11–16]. Other studies recommend specific interventions in someone with a positive MRD-test such as a haematopoietic cell transplant or immune therapy such as chimaeric antigen receptor (CAR)-T-cells. Whether such interventions reduce leukaemia relapse risk in someone with a positive MRD-test can only be proved in a randomized controlled trial [8, 17].

Most MRD-tests focus on detecting a leukaemia-related or -specific immune phenotype, cytogenetic and/or molecular abnormality [1, 2, 18–25]. A perfect MRD-test would precisely quantify only leukaemia cells biologically capable of causing leukaemia relapse and likely to do so within a defined interval after accounting for competing causes of therapy-failure [7, 8]. Routine clinical use of MRD-testing requires refinements and standardization/harmonization of assay platforms and result reporting [1, 2, 21–23].

There is consensus a flow cytometry-based MRD-test should be reproducible at a limit of detection (LoD) of ≤0.01% leukaemia cells in a blood or bone marrow sample [26]. Based on this reasoning it is proposed a multi-parameter flow cytometry (MPFC)-based MRD-test should only be declared positive if ≥ $5\times 10{{\mbox{E+5}}}$ cells are analysed and if ≥20 or ≥50 cells are positive [27–30]. However, this definition is often unmet in clinical practice. For example, modern MRD-directed, risk-stratified approach to treating childhood acute lymphoblastic leukaemia (ALL) requires an MPFC-based MRD-test done in bone marrow aspirate 2–3 weeks after starting induction chemotherapy, a time when collecting > $5\times 10{{\mbox{E+5}}}$ bone marrow mononuclear cells is difficult [31, 32]. The same limitation operates in adults receiving intensive induction chemotherapy. How should a physician use results of MRD-testing in these settings?

## Tyranny of sampling error

Assume in an MPFC-based MRD-test $N$ cells are analysed out of which $n$ cells are identified as *leukaemia cells*. By *leukaemia cells* we mean cells with immune phenotype of the leukaemia, not necessarily cells able to cause relapse within a defined interval. The conventional way to estimate MRD is ${{{{{{\rm{MRD}}}}}}}_{{{{{{\rm{conventional}}}}}}}=\frac{n}{N}$ [33, 34].

When the true proportion of leukaemia cells (“true MRD”) is < $\frac{1}{N}$, the standard error of ${{{{{{\rm{MRD}}}}}}}_{{{{{{\rm{conventional}}}}}}}$ has a magnitude even larger than true MRD because of sampling error (Supplementary Methods). Simply put, the ${{{{{{\rm{MRD}}}}}}}_{{{{{{\rm{conventional}}}}}}}$ test can be very imprecise.

To better appreciate the tyranny of sampling error consider the hypothetical example of a haematologist reviewing the following MRD-test result: $N=50000$ and $n=0$. Analysing these few cells is not uncommon in practice for reasons we discussed above. Using the conventional approach to quantifying MRD the haematologist interprets this MRD-test as ${{{{{{\rm{MRD}}}}}}}_{{{{{{\rm{conventional}}}}}}}=\frac{0}{50000}=0 \% .$ In doing so the haematologist fails to appreciate the result of this MRD-test is compatible with a broad range of true MRD values. In reality, the haematologist can only conclude MRD-test result is $\le\!\!0.006 \%$ with a 5-percent probability true MRD is actually $> 0.006 \%$.

Using Bayesian reasoning, the worst-case (probability <0.05)^1^ scenario estimate of MRD, which we denote as ${{{{{{\rm{MRD}}}}}}}_{{{{{{\rm{worst}}}}}}\_{{{{{\rm{case}}}}}}}$, can be computed using a beta distribution (the formula is “BETA.INV (0.95, $1+n$, $1+N-n$)” in Microsoft Excel; Supplementary Methods) [35].

Table 1 displays the extent to which ${{{{{{\rm{MRD}}}}}}}_{{{{{{\rm{conventional}}}}}}}$ under-estimates true MRD at different values of $N$ in the worst-case scenario (that is, by how much ${{{{{{\rm{MRD}}}}}}}_{{{{{{\rm{conventional}}}}}}}$ under-estimates ${{{{{{\rm{MRD}}}}}}}_{{{{{{\rm{worst}}}}}}\_{{{{{\rm{case}}}}}}}$). Note that when ${{{{{{\rm{MRD}}}}}}}_{{{{{{\rm{conventional}}}}}}}$ is $\le 0.01 \%$ ${{{{{{\rm{MRD}}}}}}}_{{{{{{\rm{worst}}}}}}\_{{{{{\rm{case}}}}}}}$ is considerably larger than ${{{{{{\rm{MRD}}}}}}}_{{{{{{\rm{conventional}}}}}}}$ across a broad range of $N$ values. Conversely, when ${{{{{{\rm{MRD}}}}}}}_{{{{{{\rm{conventional}}}}}}}$ is $\ge 0.1 \%$ ${{{{{{\rm{MRD}}}}}}}_{{{{{{\rm{worst}}}}}}\_{{{{{\rm{case}}}}}}}$ is usually very close to ${{{{{{\rm{MRD}}}}}}}_{{{{{{\rm{conventional}}}}}}}$ unless the number of analysed cells *N* is < 10E+5.

**Table 1:** To what extent ${{{{{{\rm{MRD}}}}}}}_{{{{{{\rm{conventional}}}}}}}$ under-estimates true MRD at different numbers of analysed cells *N* in the worst-case scenario.

		MRD_conventional_				
		10%	1%	0.1%	0.01%	0.002%
*N*	50000	−2%	−7%	−21%	−52%	−79%
	100000	−2%	−5%	−15%	−41%	−68%
	200000	−1%	−4%	−11%	−31%	−56%
	300000	−1%	−3%	−9%	−26%	−49%
	400000	−1%	−3%	−8%	−23%	−45%
	500000	−1%	−2%	−7%	−21%	−41%
	600000	−1%	−2%	−7%	−19%	−38%
	700000	−1%	−2%	−6%	−18%	−36%
	800000	−1%	−2%	−6%	−17%	−34%
	900000	−1%	−2%	−5%	−16%	−33%
	1000000	0%	−2%	−5%	−15%	−31%

Typically result of an MRD-test is interpreted as positive or negative based on applying a cut-off threshold to ${{{{{{\rm{MRD}}}}}}}_{{{{{{\rm{conventional}}}}}}}$. Our analysis of the adverse impact of sampling error (Table 1) suggests any cut-off threshold <0.01% used in ${{{{{{\rm{MRD}}}}}}}_{{{{{{\rm{conventional}}}}}}}$ would yield unreliable results with many false-negatives. Moreover, when estimating the hazard function of ${{{{{{\rm{MRD}}}}}}}_{{{{{{\rm{conventional}}}}}}}$ for leukaemia relapse risk false-negatives would cause “flattening” of the estimated curve because the contrast between MRD-positives and -negatives is attenuated by contamination of false-negative MRD-test results.

## Borrowing lessons from decision science

How to solve this problem when an inaccurate false-negative test result could have adverse clinical consequences? We propose the haematologist should instead rely on ${{{{{{\rm{MRD}}}}}}}_{{{{{{\rm{worst}}}}}}\_{{{{{\rm{case}}}}}}}$ rather than ${{{{{{\rm{MRD}}}}}}}_{{{{{{\rm{conventional}}}}}}}$ to estimate relapse risk.

Our reasoning follows. When interpreting an MRD-test result to predict relapse the haematologist is essentially playing a “chess game against *nature*”. It’s his/her 1st move to make, declaring the MRD-test result positive or negative. In response the opponent (*nature*) has two possible moves, causing relapse or not. When ${{{{{{\rm{MRD}}}}}}}_{{{{{{\rm{worst}}}}}}\_{{{{{\rm{case}}}}}}}$ is larger the haematologist is more likely to later regret if he/she declares the MRD-test result negative, because more plausibly *nature* would *play tricks* on the haematologist by causing relapse.

Ranking of people’s test results based on ${{{{{{\rm{MRD}}}}}}}_{{{{{{\rm{worst}}}}}}\_{{{{{\rm{case}}}}}}}$ from high to low values minimises the sum of regrets in the worst-case scenario because people whose MRD-test results are more likely to cause regret in case of a negative interpretation are already considered to have a higher risk of relapse. In the language of decision science, ${{{{{{\rm{MRD}}}}}}}_{{{{{{\rm{worst}}}}}}\_{{{{{\rm{case}}}}}}}$ is a *minimax regret* approach to quantifying MRD test results according to Leonard Savage’s theory of statistical decision or Herbert Simon’s theory of rational choice under uncertainty [36, 37].

## A clinical example

To illustrate using ${{{{{{\rm{MRD}}}}}}}_{{{{{{\rm{worst}}}}}}\_{{{{{\rm{case}}}}}}}$ to interpret test results we interrogated data from 883 consecutive children with ALL <16 years (Supplementary Fig. 1; Supplementary Table 1; and Supplementary Methods). The subjects were treated on the Chinese Children’s Cancer Group study ALL-2015 (CCCG-ALL-2015) protocol [32]. 618 (70%) and 265 (30%) of the children were low- and intermediate-risk at diagnosis according to the CCCG-ALL-2015 criteria. MPFC-based MRD-testing was done on bone marrow samples 19 days after starting therapy. Median number of analysed cells ($N$) was 4 × 10E+5 (Interquartile Range [IQR], 2.4–5.0 × 10E+5; Range, 3.4 × 10E+3 to 1.0 × 10E+6). 686 (78%) MRD-tests analysed <5 × 10E+5 cells, a threshold stipulated by guideline for good laboratory practice (GLP) [27, 28, 30].

**Fig. 1:**
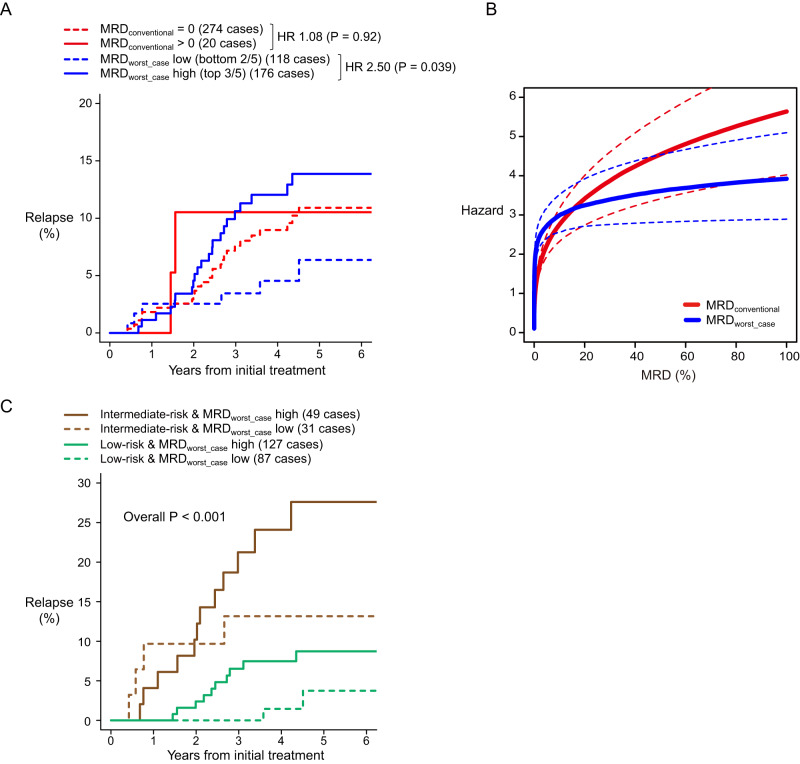
Using MRD_worse_case_ in a cohort of children with ALL. **A** Risk-stratifications based on ${{{{{{\rm{MRD}}}}}}}_{{{{{{\rm{conventional}}}}}}}$ vs. ${{{{{{\rm{MRD}}}}}}}_{{{{{{\rm{worst}}}}}}\_{{{{{\rm{case}}}}}}}$ on day 19 when ${{{{{{\rm{MRD}}}}}}}_{{{{{{\rm{conventional}}}}}}} < 0.01 \%$. Cut-off threshold for distinguishing “${{{{{{\rm{MRD}}}}}}}_{{{{{{\rm{worst}}}}}}\_{{{{{\rm{case}}}}}}}$ high” and “${{{{{{\rm{MRD}}}}}}}_{{{{{{\rm{worst}}}}}}\_{{{{{\rm{case}}}}}}}$ low” is 7.3 × 10E−6 or 0.00073%. **B** Hazard functions of ${{{{{{\rm{MRD}}}}}}}_{{{{{{\rm{conventional}}}}}}}$ and ${{{{{{\rm{MRD}}}}}}}_{{{{{{\rm{worst}}}}}}\_{{{{{\rm{case}}}}}}}$ on day 19 for relapse risk. Curve estimation is based on data from the entire cohort of 883 children. Dotted lines indicate 95-percent confidence intervals. **C** Risk-stratification based on joint consideration of estimated relapse risk at diagnosis and ${{{{{{\rm{MRD}}}}}}}_{{{{{{\rm{worst}}}}}}\_{{{{{\rm{case}}}}}}}$ on day 19 when ${{{{{{\rm{MRD}}}}}}}_{{{{{{\rm{conventional}}}}}}} < 0.01 \%$. Hazard ratios (HRs) and *P*-values are based on the Fine-Gray and Gray methods [48, 49].

294 (33%) children had ${{{{{{\rm{MRD}}}}}}}_{{{{{{\rm{conventional}}}}}}} < 0.01 \%$ on day 19, 274 (93%) of whom had zero values (*i.e*. no leukaemia cell was detected [$n=0$]). The remainder (20 [7%]) had 8–24 leukaemia cells detected. Because most children with ${{{{{{\rm{MRD}}}}}}}_{{{{{{\rm{conventional}}}}}}} < 0.01 \%$ had no leukaemia cells detected in the sample, ${{{{{{\rm{MRD}}}}}}}_{{{{{{\rm{conventional}}}}}}}$ could not identify relative relapse risk in these children. The C-statistic (the probability of pairwise agreement with relapse time [38]) of ${{{{{{\rm{MRD}}}}}}}_{{{{{{\rm{worst}}}}}}\_{{{{{\rm{case}}}}}}}$ (0.57) was significantly higher (*P* <0.001; 2-sided Wilcoxon test on 500 bootstrap samples [39]) compared with C-statistic of ${{{{{{\rm{MRD}}}}}}}_{{{{{{\rm{conventional}}}}}}}$ (0.50). In short, ${{{{{{\rm{MRD}}}}}}}_{{{{{{\rm{worst}}}}}}\_{{{{{\rm{case}}}}}}}$ was a better predictor of relapse than ${{{{{{\rm{MRD}}}}}}}_{{{{{{\rm{conventional}}}}}}}$ when ${{{{{{\rm{MRD}}}}}}}_{{{{{{\rm{conventional}}}}}}}$ was close to zero (Fig. 1A). In contrast, for the 589 (67%) children who had ${{{{{{\rm{MRD}}}}}}}_{{{{{{\rm{conventional}}}}}}}\ge 0.01 \%$ on day 19, C-statistics of ${{{{{{\rm{MRD}}}}}}}_{{{{{{\rm{worst}}}}}}\_{{{{{\rm{case}}}}}}}$ (0.58) and ${{{{{{\rm{MRD}}}}}}}_{{{{{{\rm{conventional}}}}}}}$ (0.58) were similar (*P* = 0.61).

We estimated non-linear hazard functions of ${{{{{{\rm{MRD}}}}}}}_{{{{{{\rm{conventional}}}}}}}$ and ${{{{{{\rm{MRD}}}}}}}_{{{{{{\rm{worst}}}}}}\_{{{{{\rm{case}}}}}}}$ for relapse by fitting restricted cubic spline curves using Markov chain Monte Carlo [40–43]. Since ${{{{{{\rm{MRD}}}}}}}_{{{{{{\rm{worst}}}}}}\_{{{{{\rm{case}}}}}}}$ is always larger than ${{{{{{\rm{MRD}}}}}}}_{{{{{{\rm{conventional}}}}}}}$, all else being equal, switching from ${{{{{{\rm{MRD}}}}}}}_{{{{{{\rm{conventional}}}}}}}$ to ${{{{{{\rm{MRD}}}}}}}_{{{{{{\rm{worst}}}}}}\_{{{{{\rm{case}}}}}}}$ should induce a *right-shift* of the hazard function curve. Instead, we observed the hazard function of ${{{{{{\rm{MRD}}}}}}}_{{{{{{\rm{worst}}}}}}\_{{{{{\rm{case}}}}}}}$ rose more steeply than the hazard function of ${{{{{{\rm{MRD}}}}}}}_{{{{{{\rm{conventional}}}}}}}$ (Fig. 1B). Inaccuracies in MRD-estimation using the conventional approach distorted the critical range of MRD for discriminating low- from high-risks of cumulative incidence of relapse (CIR).

Combining ${{{{{{\rm{MRD}}}}}}}_{{{{{{\rm{worst}}}}}}\_{{{{{\rm{case}}}}}}}$ on day 19 with estimated relapse risk at diagnosis further improved risk-stratification of the children whose ${{{{{{\rm{MRD}}}}}}}_{{{{{{\rm{conventional}}}}}}}$ on day 19 was $< 0.01 \%$ with a C-statistic of 0.73. This was significantly better than using ${{{{{{\rm{MRD}}}}}}}_{{{{{{\rm{worst}}}}}}\_{{{{{\rm{case}}}}}}}$ alone (0.73 vs. 0.57 [*P* < 0.001; 2-sided Wilcoxon test on 500 bootstrap samples]) or using relapse risk at diagnosis alone (0.73 vs. 0.68 [*P* < 0.001]; Fig. 1C). 214 children (73%) with ${{{{{{\rm{MRD}}}}}}}_{{{{{{\rm{conventional}}}}}}} < 0.01 \%$ on day 19 were low-risk at diagnosis and all subsequently received low-intensity therapy. The remainder (80 [27%]) were intermediate-risk at diagnosis and all received high-intensity therapy. Consequently, therapy-intensity did not confound results within each therapy cohort.

Interestingly, point-estimates for relapse at 1.5 years for high- and low-${{{{{{\rm{MRD}}}}}}}_{{{{{{\rm{worst}}}}}}\_{{{{{\rm{case}}}}}}}$ cohorts were similar and their relapse curves only diverged after 1.5 years (Fig. 1A, C). Because ${{{{{{\rm{MRD}}}}}}}_{{{{{{\rm{worst}}}}}}\_{{{{{\rm{case}}}}}}}$ corrected for (probable) under-sampling of leukaemia cells at therapy start this divergence likely resulted from expansion of pre-existing sub-clones during and/or after the end of low-intensity maintenance therapy (54 to 125 weeks) [32].

## Is MRD_worst_case_ an *index* or a *metric* for MRD?

*Index* is defined as *a number (such as a ratio) derived from a series of observations and used as an indicator or measure*. *Metric* is defined as *a standard of measurement*. Some may argue ${{{{{{\rm{MRD}}}}}}}_{{{{{{\rm{worst}}}}}}\_{{{{{\rm{case}}}}}}}$ is an *index* for MRD whilst ${{{{{{\rm{MRD}}}}}}}_{{{{{{\rm{conventional}}}}}}}=\frac{n}{N}$ is a *metric*. The distinction between *index* and *metric* is in some measure semantic. Even ${{{{{{\rm{MRD}}}}}}}_{{{{{{\rm{conventional}}}}}}}$ is a statistical construct for estimating likelihood of relapse. ${{{{{{\rm{MRD}}}}}}}_{{{{{{\rm{conventional}}}}}}}$ is what statisticians call a maximum-likelihood estimate, which is *not* the same as an estimate for the median *(i.e*. 50th-percentile) value among all the possible values of true MRD conditional on test result (Supplementary Methods). When ${{{{{{\rm{MRD}}}}}}}_{{{{{{\rm{conventional}}}}}}}$ is zero ${{{{{{\rm{MRD}}}}}}}_{{{{{{\rm{conventional}}}}}}}$ is actually the 0th-percentile (*i.e*. the lowest possible) value among all the possible values of true MRD conditional on test result! ${{{{{{\rm{MRD}}}}}}}_{{{{{{\rm{worst}}}}}}\_{{{{{\rm{case}}}}}}}$, on the other hand, is the 95th-percentile value among all the possible values of true MRD conditional on test result.

## Discussion

In this Perspective we argue the consensus GLP of MRD-testing is sub-optimal in many instances. Under these circumstances ${{{{{{\rm{MRD}}}}}}}_{{{{{{\rm{conventional}}}}}}}$ test results are sometimes mis-leading. Our analyses of data from a large cohort of childhood ALL indicates the *minimax regret* approach (${{{{{{\rm{MRD}}}}}}}_{{{{{{\rm{worst}}}}}}\_{{{{{\rm{case}}}}}}}$) improves relapse risk prediction over the current method (${{{{{{\rm{MRD}}}}}}}_{{{{{{\rm{conventional}}}}}}}$). ${{{{{{\rm{MRD}}}}}}}_{{{{{{\rm{worst}}}}}}\_{{{{{\rm{case}}}}}}}$ corrects for variation in strength of evidence in MRD-tests when predicting leukaemia relapse. Moreover, non-linear modeling of ${{{{{{\rm{MRD}}}}}}}_{{{{{{\rm{worst}}}}}}\_{{{{{\rm{case}}}}}}}$ hazard function uncovers the critical range of MRD wherein the risk of leukaemia relapse accelerates. Because the true hazard function curve is steeper and operates at a lower range of MRD than previously realised based on ${{{{{{\rm{MRD}}}}}}}_{{{{{{\rm{conventional}}}}}}}$ it is important to continue developing and using increasingly sensitive (and specific) assays for detecting residual leukaemia cells.

We acknowledge several limitations. Our analyses of the clinical data were retrospective and subject to bias. We focused on MPFC, which enumerates mostly live cells one-by-one and is distinct from other types of assays such as quantitative real time polymerase chain reaction (RT-qPCR) or next generation sequencing (NGS). We also did not analyse false-positive errors in MRD-tests, which are more likely a *biological* than statistical issue as many or perhaps most false-positives are caused by not knowing which leukaemia cells have the biological ability to cause relapse within an observation interval [44–46]. In MPFC some aberrant leukaemia phenotypes may be more confidently identified as *positive* compared with others. Consequently, further refinement of results of MRD-testing is possible. Also, molecular tests such as NGS may increase accuracy of identifying residual leukaemia cells [8, 47]. However, sampling error remains an inherent limitation for any MRD-test as does the current inability to identify leukaemia cells biologically able to cause relapse regardless of detection technology.

We suggest our proposed metric ${{{{{{\rm{MRD}}}}}}}_{{{{{{\rm{worst}}}}}}\_{{{{{\rm{case}}}}}}}$ will help haematologists more accurately predict leukaemia relapse. It is possible to further improve accuracy of predicting leukaemia relapse by considering additional data beyond MRD-tests provided confounding *predictive* and *prognostic* co-variates are adjusted for and the therapy regimen is considered.

### Supplementary information

Supplement material

## Data Availability

Clinical data are available upon reasonable request to the corresponding authors.

## Footnotes

1 Strictly speaking, the worst possible value of true MRD is always ≈1 even when ${{{{{{\rm{MRD}}}}}}}_{{{{{{\rm{conventional}}}}}}}=0$. (The chance of true MRD ≈1 might be practically zero but the probability of this unlikely event is never zero.) Defining the worst-case scenario estimate as “not likely (probability ≤ 0.05) to exceed this value” is more useful for comparing MRD-test results.
