# Supplementary material for: Have we been qualifying measurable residual disease correctly?
Source: Leukemia. 2023 Sep 13;37(11):2168–72. doi: 10.1038/s41375-023-02026-4 (PMC10624632; doi:10.1038/s41375-023-02026-4)
Supplement: Supplementary file 1 — Supplement material [file 41375_2023_2026_MOESM1_ESM.docx]

**Supplement Material**

**Methods**

**Subjects**

We interrogated data from 883 consecutive children < 16 years with acute lymphoblastic leukaemia (ALL) seen at the Institute of Hematology, Chinese Academy of Medical Sciences (Tianjin, China) during 2015 – 2020. Subjects were treated on the Chinese Children's Cancer Group study ALL-2015 (CCCG-ALL-2015) protocol [1].  Subjects were classified as *low-risk* at diagnosis if they had B-cell ALL (B-ALL); did not have hypo-diploidy (< 44 chromosomes), t(1;19), t(9;22), *KMT2A* rearrangement, or intra-chromosomal amplification of chromosome 21 (iAMP21); did not have CNS 3 state or testicular leukaemia; and furthermore fit ≥ 1 of the following 3 criteria: (1) age 1 – 10 years and WBC ≤ 50 × 10E+9/L; (2) hyper-diploidy (≥ 50 chromosomes); (3) with a *ETV6::RUNX1* fusion. Otherwise subjects were classified as *intermediate-risk* at diagnosis. A CONSORT flow diagram is displayed in **Supplement Figure 1**. Co-variates of the subjects are available in **Supplement Table 1**. The last follow-up was 15 May 2023.

**Quantification of measurable residual disease**

MRD was quantified by multi-parameter flow cytometry (MPFC; BD FACSCanto Plus flow cytometer [V657338000225; BD Biosciences, San Jose, CA, USA] with BD FACSDiva Software v8.0.1) analyses of bone marrow. All subjects in the study cohort had MRD-test data available 19 days after starting therapy.

For B-ALL we used antibodies to CD19 (PE-Cy7 IM3628; Beckman Coulter, Brea, CA, USA), CD10 (PE A07760; Beckman Coulter), CD34 (PerCP-Cy5.5 343522; BioLegend, San Diego, CA, USA), CD20 (APC-H7 641396; BD Biosciences), CD22 (BV421 302524; BioLegend), CD38 (FITC A07778; Beckman Coulter), CD45 (V500 662912; BD Biosciences) and CD81 (APC 551112; BD Biosciences). For T-cell ALL (T-ALL) we used two runs of MPFC for leukaemia cell detection. In one run antibodies to terminal deoxynucleotidyl transferase (FITC F7139; Dako, Santa Clara, CA USA), cytoplasmic CD3 (Pacific Blue 558117; BD Biosciences), CD2 (PE A07744; Beckman Coulter), CD7 (APC A6005R12; QuantoBio, Beijing, China), CD10 (APC-Cy7 312212; BioLegend), CD33 (PE-Cy7 664997; BD Biosciences), CD34 (PerCP-Cy5.5 343522; BioLegend), CD45 (V500 662912; BD Biosciences) and CD117 (PE-Cy7 313212; BioLegend) were used. In the 2nd run antibodies to CD3 (PE-Cy7 737657; Beckman Coulter), CD4 (V450 560345; BD Biosciences), CD5 (PerCP-Cy5.5 665001; BD Biosciences), CD7 (FITC 664935; BD Biosciences), CD8 (APC-H7 641400; BD Biosciences), CD16 (APC 302012; BioLegend), CD45 (V500 662912; BD Biosciences), CD56 (APC 362504; BioLegend) and CD99 (PE 555689; BD Biosciences) were used. MRD was defined by either leukaemia cell-associated immune phenotype identified at diagnosis or an immune phenotype deviating from normal haematopoietic cells [2].

**Statistics**

$$\mathbf{MRD}_{\mathbf{conventional}}$$

Denote the true proportion of leukaemia cells (“true MRD”) as $p$ and of non-leukaemia cells as $q=1-p$. By *leukaemia cells* we mean cells with immune phenotype characteristic of B- or T-ALL, not necessarily cells able to cause relapse within a certain interval.

Assume $N$ cells were analysed out of which $n$ cells were “positive” (*i.e.* leukaemia cells). The conventional way to estimate MRD is $\mathrm{MRD}_{\mathrm{conventional}}=\frac{n}{N}$ [3, 4]; this expression assumes the laboratory assay has perfect sensitivity and perfect specificity and under these assumptions is a maximum-likelihood estimate that is unbiased (expected value = $p$) but imprecise (standard error = $\sqrt{\frac{pq}{N}}=p\sqrt{\frac{q}{pN}}$). When $pN<1$, standard error of $\mathrm{MRD}_{\mathrm{conventional}}$ could be even larger than the true MRD value we would like to estimate!

$$\mathbf{MRD}_{\boldsymbol{worst\_case}}$$

The likelihood function $L\left( p,q \right)=p^{n}q^{N-n}$ is binomial, and when the prior for $\left( p,q \right)$ is *non-informative* the posterior distribution of $\left( p,q \right)$ is $\mathrm{Dirichlet}\left( 1+n\text{, }1+N-n \right)$. Furthermore, the marginal posterior distribution of $p$ is $\mathrm{Beta}\left( 1+n\text{, }1+N-n \right)$ [5].

We define $\mathrm{MRD}_{worst\_case}$ to be the 95th-percentile value of $p$ according to $p$’s posterior distribution: $\mathrm{MRD}_{worst\_case}=\varphi^{-1}\left( 0.95; 1+n\text{, }1+N-n \right)$, where $\varphi^{-1}\left( \cdot\right)$ is the inverse cumulative distribution function of beta distribution. $\mathrm{MRD}_{worst\_case}$ can be computed in Microsoft Excel using an easy one-line command: BETA.INV(0.95, $1+n$, $1+N-n$).

**Cumulative incidence of relapse**

Cumulative incidence of relapse (CIR) was estimated by the Gray method, treating non-relapse mortality as a competing risk [6]. C-statistic was calculated as the probability of pairwise agreement between an MRD metric or index and relapse time [7]. Comparison of C-statistics was calculated using two-sided Wilcoxon test conducted on bootstrap samples [8].

**Nonlinear hazard function for CIR**

The non-linear hazard functions of $\mathrm{MRD}_{\mathrm{conventional}}$ and $\mathrm{MRD}_{worst\_case}$ for relapse were estimated by fitting restricted cubic spline curves using Markov chain Monte Carlo [9-12].

References:

1. Yang W, Cai J, Shen S, Gao J, Yu J, Hu S, et al. Pulse therapy with vincristine and dexamethasone for childhood acute lymphoblastic leukaemia (CCCG-ALL-2015): an open-label, multicentre, randomised, phase 3, non-inferiority trial. Lancet Oncol. 2021;22(9):1322-32.

2. Contreras Yametti GP, Ostrow TH, Jasinski S, Raetz EA, Carroll WL, Evensen NA. Minimal Residual Disease in Acute Lymphoblastic Leukemia: Current Practice and Future Directions. Cancers (Basel). 2021;13(8).

3. Theunissen P, Mejstrikova E, Sedek L, van der Sluijs-Gelling AJ, Gaipa G, Bartels M, et al. Standardized flow cytometry for highly sensitive MRD measurements in B-cell acute lymphoblastic leukemia. Blood. 2017;129(3):347-57.

4. Modvig S, Hallbook H, Madsen HO, Siitonen S, Rosthoj S, Tierens A, et al. Value of flow cytometry for MRD-based relapse prediction in B-cell precursor ALL in a multicenter setting. Leukemia. 2021;35(7):1894-906.

5. Gelman A, Carlin JB, Stern HS, Dunson DB, Vehtari A, Rubin DB. Bayesian Data Analysis. 3rd ed. Boca Raton, FL: Chapman and Hall/CRC; 2013.

6. Gray RJ. A class of K-sample tests for comparing the cumulative incidence of a competing risk. Ann Stat. 1988;16:1141-54.

7. Pencina MJ, D'Agostino RB, Sr. Evaluating Discrimination of Risk Prediction Models: The C Statistic. JAMA. 2015;314(10):1063-4.

8. Efron B. Bootstrap methods: another look at the jackknife. Ann Stat. 1979;7(1):1-26.

9. Kirkpatrick S, Gelatt CD, Jr., Vecchi MP. Optimization by simulated annealing. Science. 1983;220(4598):671-80.

10. Green PJ, Silverman BW. Nonparametric Regression and Generalized Linear Models: A Roughness Penalty Approach. London: Chapman & Hall; 1994.

11. Gauthier J, Wu QV, Gooley TA. Cubic splines to model relationships between continuous variables and outcomes: a guide for clinicians. Bone Marrow Transplant. 2020;55(4):675-80.

12. Chen J, Gale R, Feng Y, Hu Y, Qi S, Liu X, et al. Are haematopoietic stem cell transplants stem cell transplants, is there a threshold dose of CD34-positive cells and how many are needed for rapid posttransplant granulocyte recovery? Leukemia. 2023.

**Supplement Figure 1. CONSORT diagram**


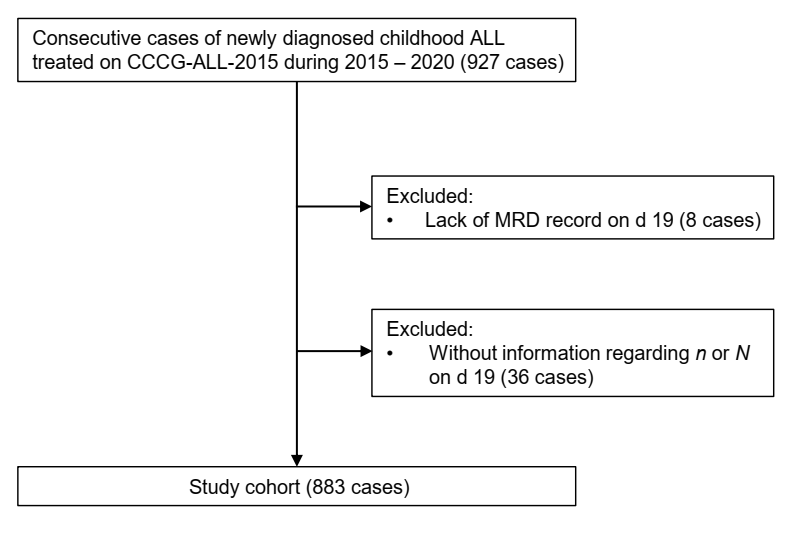


**Supplement Table 1. Co-variates of the subjects**

|  | **Study cohort (883 cases)** |
| --- | --- |
| **Year at diagnosis, cases (%)** |  |
| 2015 | 83 (9) |
| 2016 | 170 (19) |
| 2017 | 160 (18) |
| 2018 | 202 (23) |
| 2019 | 188 (21) |
| 2020 | 80 (9) |
| **Age at diagnosis, median years (range)** | 5.1 (0.6 – 15.8) |
| **Male, cases (%)** | 514 (58) |
| **Ethnicity, cases (%)** |  |
| Han | 803 (91) |
| Hui | 11 (1) |
| Manchu | 27 (3) |
| Mongol | 30 (3) |
| Others | 12 (1) |
| **Leukaemia type, cases (%)** |  |
| B-ALL | 840 (95) |
| T-ALL | 43 (5) |
| **CCCG-ALL-2015-defined relapse risk at diagnosis, cases (%)** |  |
| Low-risk | 618 (70) |
| Intermediate-risk | 265 (30) |
| **WBC count at diagnosis, median 10E+9/L (range)** | 8.2 (0.8 – 800.7) |
| **Analysed cells in MPFC done on d 19, median (range)** | 400,000 (3,400 – 1,010,000) |
| **MRD_conventional_ on d 19, median % (range)** | 0.09 (0.00 – 88.05) |
| **MRD_worst_case_ on d 19, median % (range)** | 0.10 (0.0003 – 88.13) |
| **Follow-up duration, median years (range)** | 4.5 (0.1 – 7.8) |
| **Survival, % (95% CI)** |  |
| 1 year | 98 (97 – 99) |
| 3 years | 94 (93 – 96) |
| 5 years | 92 (90 – 94) |
| **Cumulative incidence of relapse, % (95% CI)** |  |
| 1 year | 3 (2 – 5) |
| 3 years | 14 (11 – 16) |
| 5 years | 20 (17 – 22) |

Abbreviations: CI, confidence interval; WBC, white blood cells.
